# Simon’s Algorithm in the NISQ Cloud

**DOI:** 10.3390/e27070658

**Published:** 2025-06-20

**Authors:** Reece Robertson, Emery Doucet, Ernest Spicer, Sebastian Deffner

**Affiliations:** 1 Department of Physics, University of Maryland, Baltimore County (UMBC), Baltimore, MD 21250, USA; 2 Quantum Science Institute, University of Maryland, Baltimore County (UMBC), Baltimore, MD 21250, USA; 3 Department of Computer Science and Electrical Engineering, University of Maryland, Baltimore County (UMBC), Baltimore, MD 21250, USA; 4 Sagax.ai, Pullman, WA 99163, USA; 5 National Quantum Laboratory, College Park, MD 20740, USA

**Keywords:** NISQ computing, Simon’s algorithm, quantum advantage

## Abstract

Simon’s algorithm was one of the first to demonstrate a genuine quantum advantage in solving a problem. The algorithm, however, assumes access to fault-tolerant qubits. In our work, we use Simon’s algorithm to benchmark the error rates of devices currently available in the “quantum cloud”. As a main result, we objectively compare the different physical platforms made available by IBM and IonQ. Our study highlights the importance of understanding the device architectures and topologies when transpiling quantum algorithms onto hardware. For instance, we demonstrate that two-qubit operations on spatially separated qubits on superconducting chips should be avoided.

## 1. Introduction

Recent years have seen a rapid increase in investments in quantum technologies. Some market analysts even project the global volume to reach up to USD 1.3 trillion in value by the mid-2030s [1]. This unprecedented growth in economic interest is driven by the fact that fully functioning quantum devices will have the ability to exponentially outperform classical technologies. This quantum advantage originates in the peculiarities of quantum physics, which allows quantum technologies to perform certain tasks with exponentially fewer resources than would be required by any classical device [2,3,4,5].

Global excitement has been further fueled by the first experiments demonstrating quantum advantage for judiciously designed computational tasks [6,7,8,9,10,11,12]. However, currently available hardware is somewhat “small” and, more importantly, it is still prone to noise. Despite continuing progress in the development of fault-tolerant quantum computers [13], there is a consensus that we are currently in the era of noisy, intermediate-scale quantum (NISQ) devices [14,15,16,17,18,19,20,21,22,23,24,25,26]. This raises the natural question of how these NISQ devices perform when tested with genuinely quantum algorithms that were explicitly designed to exhibit quantum advantage.

Arguably, the most famous quantum algorithm is due to Shor [27], which was the first to demonstrate potential applications of quantum computing to a “practical” problem—namely, the factorization of an integer into prime numbers. Remarkably, the first experimental implementations were already reported almost three decades ago [28], albeit at very small scales. Similar experiments have investigated the performance of random circuit sampling [29] and Grover’s search and the Bernstein–Vazirani algorithms [30]. Importantly, it was concluded that fair sampling has not yet been achieved on any of the devices by IBM, Rigetti, IonQ, and D-Wave [31].

In the present paper, we use Simon’s algorithm [32] to benchmark several NISQ devices available for remote, cloud access. This algorithm is significant since it was among the first to use the quantum Turing machine framework to obtain a provable exponential speedup over its classical probabilistic counterpart [2]. In other words, it was among the first to show that a quantum Turing machine can violate the Church–Turing thesis [33]. The noise resilience of Simon’s algorithm has been tested by performing Monte Carlo simulations of stochastic Pauli noise operators [34], showing that the algorithm is rather susceptible to imperfections and noise. Hence, we propose to employ the performance of the algorithm as a very sensitive tool for noise diagnostics of NISQ hardware.

We have implemented two versions of Simon’s algorithm, with oracles chosen to require the minimal and maximal number of entangling operations (ignoring oracles that include permutations); the instances were run on three versions of a superconducting platform available at IBM and three trapped-ion devices by IonQ. As a measure of performance, we computed the percentage of trials that returned an invalid answer at each problem size, a metric we refer to as the *algorithmic error rate*. For each physical platform, we compared the resulting error rate with the prediction of the noisy simulators provided by the quantum computing companies.

As a main result, we found that all the NISQ computers examined experienced an increase in algorithmic error as the problem size increased. The error rate scaled approximately linearly for the IonQ devices, whereas it exhibited a stark departure from linear scaling on the IBM devices for the most complex algorithm. We traced this observation back to the presence of entangling gates between spatially separated qubits. Finally, we found that none of the noisy simulators provided for any of the tested devices captured the observed scaling quantitatively.

Taking our data as measured from each device and extrapolating, we predict that all tested devices should exhibit an error rate of 50% for problems requiring more than 50 qubits. (Depending on the details of the estimate, more than ≈53 qubits are required for the unambiguous emergence of quantum advantage [6].) This means that the algorithm completely fails for problems large enough to possibly exhibit quantum advantage, as an error rate of 50% corresponds to random guessing.

We draw two main conclusions from our results, namely (i) NISQ devices available for remote access are still too noisy to support genuine quantum advantage, and (ii) when transpiling algorithms to physical platforms, close attention needs to be paid to the QPU architecture.

## 2. Hidden Subgroups and Simon’s Problem

A good starting point in the quest for quantum advantage is the *hidden subgroup problems* [2]. In simple terms, such problems require decrypting information that is “hidden” from direct access. Typical examples include factoring, evaluating the discrete logarithm, checking graph isomorphisms, and the shortest vector problem. Arguably, the most prominent example of hidden subgroup problems is Shor’s algorithm [27], which in turn was inspired by Simon’s problem [32].

As it was originally posed, Simon’s algorithm distinguishes between two classes of functions that operate on bitstrings of size *n*. Assume that we are given a function *f*, which is either one-to-one or two-to-one with the property that there exists some “secret string” *s* such that for every two inputs that map to the same output, the XOR of the inputs is *s* [32,35]. In other words, *f* is either a one-to-one function or it is a two-to-one periodic function with period *s*. Further, assume that we have access to a black-box quantum oracle, $U_{f}$, that operates on two quantum registers |x〉 and |y〉. When applied to these registers, $U_{f}$ evaluates *f* on the contents of the first register and XORs it into the second register. In mathematical terms,

(1) $$U_{f}\left(|x\rangle |y\rangle \right)=|x\rangle |f\left(x\right)⊕y\rangle \;,$$

where ⊕ denotes XOR.

Given such a $U_{f}$, Simon’s problem is to classify the associated *f* into one of the two classes [32]. If *f* is of the second class, then the period *s* of *f* must also be identified. In the present work, we choose the provided function *f* to be of the second class, that is, *f* is two-to-one with period *s*.

**Figure 1:**
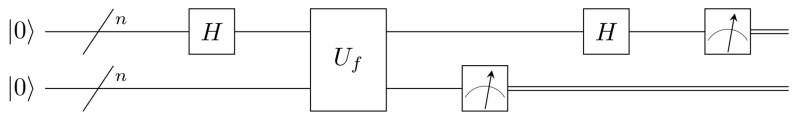
Quantum circuit diagram for Simon’s algorithm: Note the use of two quantum registers of size *n*, both initialized to the zero state |0〉, as well as the oracle $U_{f}$. The algorithm uses two Hadamard transformations and $U_{f}$ to create a superposition over all the size *n* bitstrings that are orthogonal to the secret string *s*. An iteration of the algorithm is successful if the final measurement result is indeed orthogonal to *s* (this is always true in the noise-free case). The entire algorithm is successful if a complete set of n−1 linearly independent bitstrings is measured, and the resulting system is solved classically in polynomial time.

For clarity, we depict the corresponding quantum circuit in Figure 1.

For the following analysis, it will be instructive to summarize the explicit steps of the implementation: Simon’s algorithm prepares two quantum registers, each containing *n* qubits in the ground state |0〉, before a Hadamard transformation is applied to the first register. Then, $U_{f}$ is applied, which stores the evaluation of *f* on all possible inputs in the second quantum register. The second register may then be measured to select a single element of the image of *f* (the measurement of the second register is optional; however, it simplifies the mathematics of the problem and is often included in presentations of the algorithm). If this is done, then the superposition of the entangled first register correspondingly collapses to a superposition of the two elements of the domain of *f* which map to this output. A second Hadamard transformation is then applied to the first register, and it is measured as well.

This second Hadamard transformation is a critical step of the algorithm. It creates an interference pattern on the first register, resulting in a superposition of all elements of the domain of *f*, which are orthogonal to the secret string *s*. If the algorithm has worked properly, then the measurement of the first register will always return a bitstring that is orthogonal to the secret string *s*. Which specific bitstring is returned is random, so repeating the experiment will give a different bitstring that is also orthogonal to *s* with high probability. With n−1 linearly independent bitstrings orthogonal to *s*, it is possible to construct a system of equations with sufficient information to solve for *s* in polynomial time using a classical linear solver.

Before continuing, we pause for a note on terminology. Solving Simon’s problem requires repeated iterations of a particular quantum circuit (Figure 1), followed by classically solving the resulting system of n−1 linearly independent bitstrings. The term “Simon’s algorithm” can therefore be used to reference both (1) a single iteration of the quantum circuit, or (2) repeated iterations of the quantum circuit followed by classical post-processing. Because our work is concerned with the performance of the quantum circuit on NISQ hardware, we use the terms “Simon’s algorithm” and “algorithm” in the former sense throughout this paper.

In the following, we report the performance of this implementation of Simon’s algorithm on NISQ devices. This is particularly interesting since Simon’s algorithm was one of the first to provide a provable divergence in runtime between a probabilistic Turing machine and a quantum Turing machine. Solving this problem scales exponentially on probabilistic, classical machines while scaling polynomially on quantum computers [32]. However, this speedup is predicated on the assumption that one has access to a sufficient number of fault-tolerant (noise-free) qubits. When running Simon’s algorithm on real quantum devices, it is to be expected that this is not the case. In the presence of noise, some percentage of the iterations of an instance of Simon’s algorithm will produce results that are *not* orthogonal to *x*. The main objective of our analysis is to investigate how poorly the algorithm performs on real hardware and what we can learn about the origins of the failure from the results.

## 3. The Quantum Cloud

Over the last decade, several large corporations as well as smaller start-up companies have made their NISQ devices available for cloud access. This offers exciting prospects for fundamental as well as applied research, as now with comparatively little effort “quantum experiments” can be performed. However, it has also already been demonstrated that the reported quantum volume, defined using the number of qubits and the number of operations that can be effectively implemented on the device [36], often overestimates what is experimentally accessible [37]. Other metrics such as randomized benchmarking [38] and cross-entropy benchmarking [39] have been utilized in attempts to demonstrate quantum supremacy; however, these benchmarks utilize random quantum circuits. As such, they may not capture the error that arises on nonrandom algorithms. Moreover, from the point of view of cloud users, one may be more interested in the performance of a specific algorithm, rather than full error characteristics. Thus, an exploration of error using a metric applicable to a genuine, specific algorithm is desirable. Finally, hardware-specific characteristics affect algorithm execution [40].

This raises the somewhat natural question: how do algorithms designed for the very purpose of exhibiting quantum advantage perform on available NISQ devices? For our study, we answer this question using IBM’s superconducting platform and IonQ’s trapped-ion devices. We note that many other companies have quantum computers with some level of cloud access, including D-Wave (annealing) [41], Google (superconducting) [42], Honeywell (trapped-ion) [43], Quantinuum (trapped-ion) [44], QuEra (neutral atom) [45], Rigetti (superconducting) [46], Xanadu (photonic) [10], and others. An ideal investigation of this topic would include executions on all of these devices; however, both the cost and administrative overhead required to access these devices is beyond the constraints of this project. For our IBM experiments, we made use of the free-access plan the company offers. For our IonQ experiments, we used direct access through QLab, and the total cost of all of our IonQ experiments was USD 10,216.20. Access to other hardware must be obtained through a partnership with the company or with a third-party platform like AWS, and pricing models vary.

### 3.1. Superconducting Qubits—IBM

The web application called *IBM Quantum*, formerly known as *IBM Quantum Experience*, provides cloud-based access to a variety of tutorials, simulators, and real quantum processors. The service was originally launched in May 2016, and the systems have gained some maturity over the last decade.

IBM’s quantum processors are made up of superconducting transmon qubits [47], located in dilution refrigerators at the IBM Research headquarters at the Thomas J. Watson Research Center. The original system consisted of only five qubits connected in a star geometry, which, however, already supported studies of fundamental questions in physics; see e.g., Ref. [48]. The largest generation, the Condor chip, has more than 1000 qubits [49]. This has created significant interest in the quantum community; for instance, Ref. [50] has analyzed IBM cloud data (including job time, queue time, compilation time, etc.) and compared it to classical high-performance computing cloud services.

**Figure 2:**
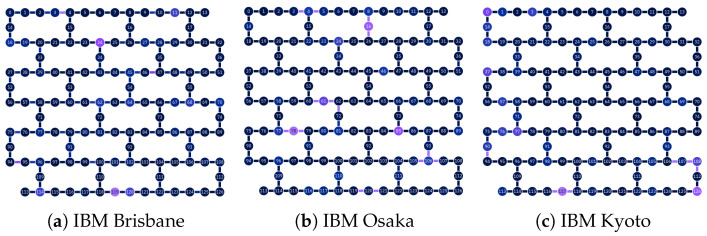
Device topologies for IBM computers [51]: The color of qubits and connections represents the single qubit readout error and ECR error, respectively, at the time the image was taken. Lighter colors correspond to higher error rates. For single qubit readout errors, the scale ranges from $3.6\times 10^{-3}$ to $3.45\times 10^{-1}$, with a median of $1.45\times 10^{-2}$. For two qubit ECR errors, the scale ranges from $2.31\times 10^{-3}$ to $1.057\times 10^{-1}$, with a median of $8.002\times 10^{-2}$.

For our work, we used the 127-qubit Brisbane, Osaka, and Kyoto superconducting quantum processors, for which noisy simulators are also provided. The device topologies for each quantum processing unit (QPU) are depicted in Figure 2. Observe that all chip topologies are identical, as these three computers are all instances of the IBM Eagle chip design.

### 3.2. Ion Traps—IonQ

**Figure 3:**
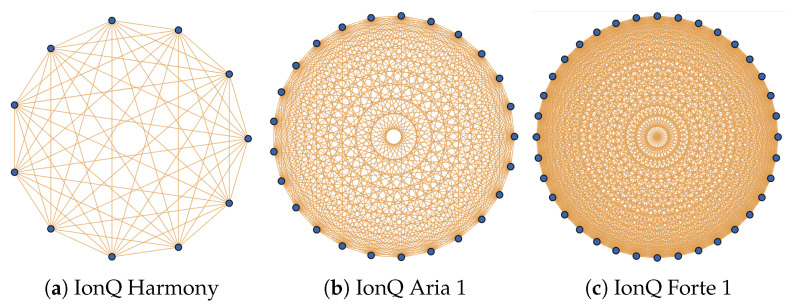
Device topologies for IonQ computers [53]: Observe that IonQ’s trapped-ion computers have full all-to-all connectivity.

A fundamentally different type of system is the trapped-ion approach by IonQ [52], which has physically located its computers in College Park, Maryland. Typically, trapped-ion NISQ devices exhibit several advantages over other platforms, such as accuracy, predictability, and coherence time [52]. However, such systems have the disadvantage that the number of available qubits is much smaller. For instance, the largest IonQ device (Forte) has only 36 qubits. With IonQ’s devices, some of the limitations arising from the small qubit number are made up by the fact that they possess full connectivity. In Figure 3, we depict the topologies of IonQ’s 11-qubit Harmony, 25-qubit Aria, and 36-qubit Forte devices.

The details of the physical parameters of all devices can be found in Appendix A.

## 4. Simon’s Algorithm on NISQ

### 4.1. Implementation of the Algorithm

In the abstract formulation of Simon’s algorithm, cf. Figure 1, the oracle $U_{f}$ is an unspecified function. This is sufficient for considerations of quantum advantage, and in fact, the algorithm is formulated to yield results independent of the precise form of the oracle.

**Figure 4:**
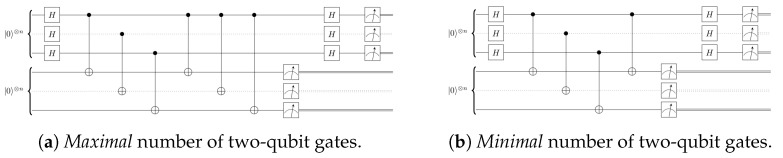
Complex and simple oracle for Simon’s algorithm. Each dotted wire represents (n−2) qubits, and all operations on dotted wires are repeated following the pattern across all qubits.

However, to implement the algorithm on real hardware, $U_{f}$ clearly must be specified. On NISQ devices, every gate operation is accompanied by its native noisiness, and hence one would expect the performance of the entire algorithm to be dependent on the explicit choice of $U_{f}$. Luckily, Simon’s algorithm is simple enough that one can easily identify the “worst- and best-case scenarios”. The worst-case scenario corresponds to the value of *s* which induces a $U_{f}$ with the *maximal* number of two-qubit operations, and the best-case scenario corresponds to the value of *s* which is representable with the *minimal* number of two-qubit gates. For brevity, we refer to these extreme cases as “complex” and “simple” oracles. The circuits that implement Simon’s algorithm with these two oracles are shown in Figure 4.

We note that in general, an oracle can include a permutation of the basis states that requires additional two-qubit operations beyond our “worst-case scenario”. We did not consider such permutations in this work, as the foundational task of implementing an oracle without a permutation is sufficiently difficult for current hardware, and this problem must be resolved before oracles involving permutations can be attempted.

That settled, the algorithm was implemented as follows: First, we defined a function *f* on bitstrings of size *n*, where *s* was the string of *n* 1 s in the complex case, and the string of n−1 0 s preceded by a 1 in the simple case. Then, we constructed the corresponding oracle $U_{f}$ for the complex and simple cases. Next, we allocated two registers of *n* qubits on the device. As depicted in Figure 4, we applied a Hadamard transform to the first register, then the function $U_{f}$ to the first and second register, and then a second Hadamard transform to the first register. We concluded the algorithm by measuring all qubits to obtain an output bitstring. We extracted the bitstring corresponding to the first register and then computed its product with *s*. Finally, we repeated the algorithm for many shots, recording the percentage of shots that yielded invalid measurement results, i.e., bitstrings not orthogonal to *s*. To quantify the performance of the algorithm, we defined the *algorithmic error rate* to be the percentage of results not orthogonal to *s*.

### 4.2. Results on IBM Devices

Our experiments on the real IBM quantum devices, Brisbane, Osaka, and Kyoto, were submitted through the Composer tool of IBM’s Quantum web application (https://quantum.ibm.com/composer, accessed on 11 June 2024). This tool provides a graphical user interface through which one can create quantum circuit diagrams and submit them for execution on hardware. It also automatically transpiles algorithms written agnostically of hardware into a format that runs on a particular backend. Transpiliation is a heuristic process that utilizes the current calibration of the machine [54], which means that the gate operations utilized and physical qubits selected often vary between submissions of the algorithm.

We repeated each experiment three times and averaged the results. In each experiment, *n* ranged from 2 to 12, which meant a total of 33 jobs were executed on each device with 8192 shots per job. As alluded to above, we observed that the algorithm was transpiled differently for different runs of the same instance. For example, our second run of the n=12 complex oracle utilized qubits 5–9 on IBM Osaka, while our third run of the same algorithm on the same device did not. We chose to allow for this variation in the physical implementation of the algorithm as it allowed the IBM system to dynamically optimize for the current characterization of the device. Hence, all of our experiments were as near as possible to the most efficient implementation of the algorithm on each device at the time of execution.

We also conducted experiments on the noisy simulators provided for the devices. To this end, we utilized the IBM AER local simulator, seeded using error models derived from the real devices, including single-qubit gate errors, two-qubit gate errors, and single-qubit readout errors [55]. To generate the data for these noisy simulators, we followed the same process as described above, with the following two modifications: Firstly, we repeated the entire process thirty times instead of three; secondly, we obtained the latest noise characteristic calibration data from the IBM API interface between each iteration of the experiment (i.e., the same model was used for n∈[2,12], but a new model was recomputed before *n* reset).

**Figure 5:**
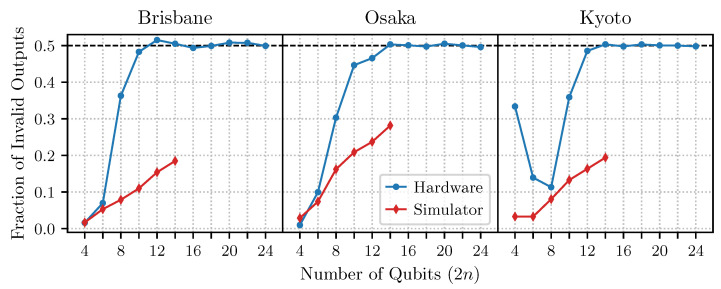
Simon’s algorithm was performed for the complex oracle at various sizes on IBM devices and simulators, cf. Figure 4a. By ∼12 qubits, the real device hovers around 50% algorithmic error, which is indistinguishable from randomly guessing solutions to the problem from the space of all possibilities. See Appendix B for results obtained after a major Qiskit update released in May 2024.

**Figure 6:**
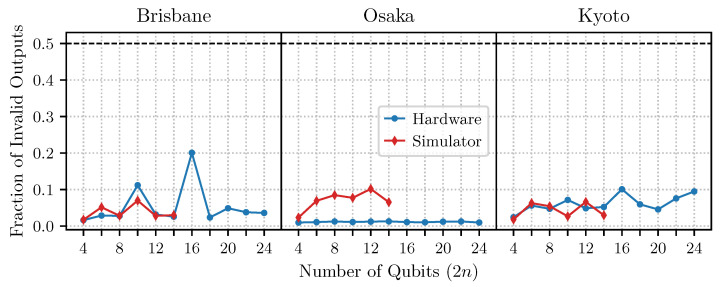
Simon’s algorithm performed for the simple oracle at various sizes on IBM devices and simulators, cf. Figure 4b.

Figure 5 shows our results for the complex oracle requiring the maximal number of two-qubit gates, and in Figure 6, we present the results for the simplest oracle. We observe that already for moderate sizes of the problem, n>8, the more complicated oracle leads to a failure of the algorithm. Interestingly, the simplest oracle performs remarkably well for all problem sizes.

More curious is the output of the noisy simulators. While for the simpler case in Figure 6, the output of the simulator and the hardware are qualitatively consistent, this is not the case for the more complicated oracle. In this case, the AER local simulators predict a roughly linear scaling in algorithmic error as the problem size increases for all devices, while our experimental results all show a large jump in error around n=4.

A plausible hypothesis is that this increase in error coincides with the addition of several swap gates into the algorithm, as the chip layout cannot provide all the needed direct connections for problems of this size. Interestingly, however, the simulated algorithm also inserts swap gates of the same pattern, but the jump in error is not observed. Moreover, gate count scales roughly linearly as a function of problem size, meaning that error does not arise from dramatic changes to algorithm complexity or runtime. Thus, the dramatic jump in algorithmic error observed on the hardware may be due to correlated errors that accompany the addition of the swap operations, which are not captured in the simulator’s noise model.

**Figure 7:**
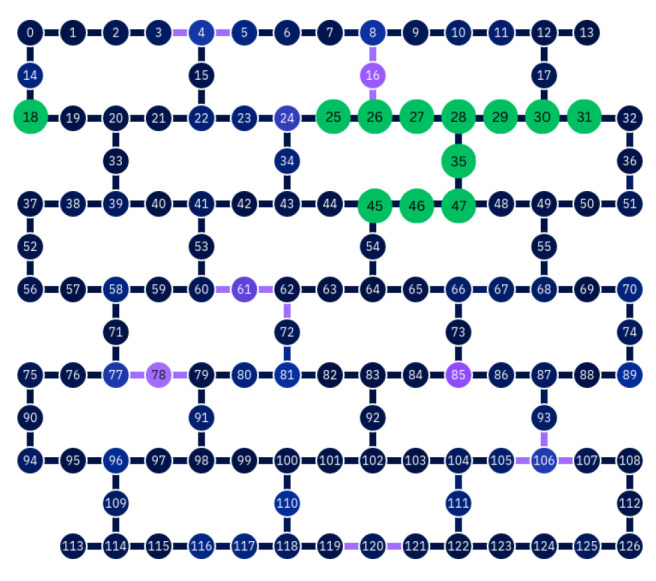
The layout of a transpiled circuit implementing Simon’s algorithm with the complex oracle for n=5 on IBM Osaka. Active qubits are marked in green, and unused qubits are depicted in blue or purple. As in Figure 2, the color of the unused qubits indicates the magnitude of readout error present during the device calibration, with lighter values indicating more error. It is worth noting that all used qubits marked in green originally appeared in dark blue, indicating comparatively little readout error. Note that after transpiliation, qubit 18, although active, is idle for the duration of the algorithm and does not interact with the other active qubits. All other active qubits interact directly with one or two other qubits via entangling operations.

This hypothesis is further corroborated by inspecting the layout of the transpiled quantum circuits. In Figure 7, we depict one example for n=5 on IBM Osaka, in which we have marked the “active” qubits in the QPU. We notice that spatially separated qubits are active, which necessitates the physical implementation of two-qubit gates across substantial portions of the QPU. The natural question arises to what degree the two-qubit gate error increases with the spatial separation between the qubits.

**Figure 8:**
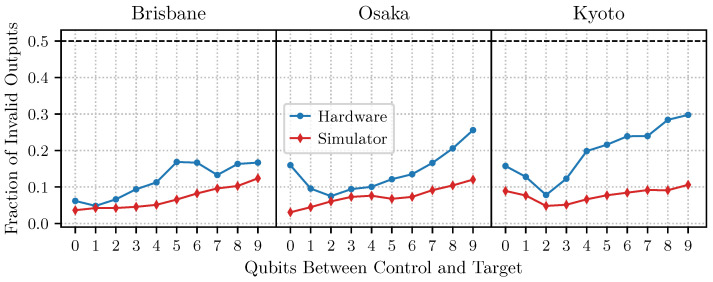
Failure of CNOT gates as a function of spatial separation. Before compilation, the control bit was selected to be qubit 39 for each experiment, and the target bit ranged from bit 40 to 49 (cf. Figure 2 and Figure 7).

In Figure 8, we collect our findings for the error rates of the CNOT gate as a function of the spatial separation of the control and target qubits. As expected, the error rate grows as a function of distance, but we also observe that the noisy simulators underestimate this effect. While it cannot be excluded that there are further hardware and software issues that contribute to the failure of Simon’s algorithm with the most complicated oracle, we can conclude that two-qubit operations between spatially separated qubits are a significant source of error.

Finally, we reiterate that, after about 10 qubits, the algorithmic error observed for the complex oracle on the real hardware is approximately 50%. This indicates a complete failure of Simon’s algorithm. Randomly outputting bitstrings sampled uniformly from the space of all bitstrings of size *n* would yield an error rate of approximately 50%, which is indistinguishable from the results observed here.

### 4.3. Results on IonQ Devices

For the IonQ trapped-ion devices, our experiments were submitted via the IonQ APIs using a hardware-agnostic Qiskit program before being transpiled for each IonQ device. Note that this implies that the actual sequence of gates physically implemented may have varied for each job performed. For IonQ Harmony, we repeated our experiment twice, with n∈[2,5] and 8192 shots per value of *n*. Likewise, for IonQ Aria 1, we repeated our experiment twice, with n∈[2,12] and 8192 shots per value of *n*. On IonQ Forte, we had access to enough qubits to test n∈[2,17]; however, we only conducted our experiment once with 4096 shots per value of *n* on this device. This simplification was mandated by budget and time constraints. At the time of job execution, Forte was only available through one-hour reservations costing USD 7000.

In complete analogy to the experiments on IBM’s platforms, we made use of the cloud-based noisy simulators provided for their Harmony and Aria devices (currently, there is no noisy simulator offered for Forte). As before, we performed 30 repetitions of our experiment on these simulators with 8192 shots per value of *n*. To the best of our knowledge, IonQ does not provide information about how often the simulators are recalibrated, nor what noise characteristics are used in their construction. However, it does state that the results of their simulators are only broadly similar to the results of the physical devices [56].

**Figure 9:**
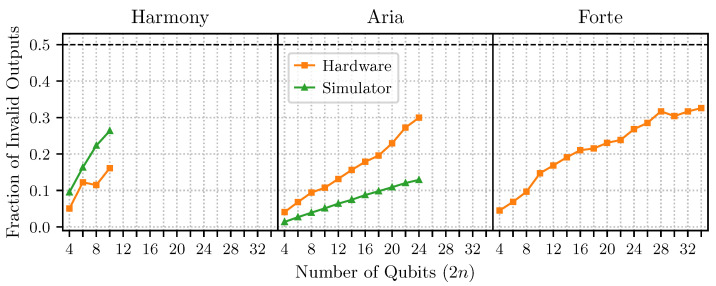
Simon’s algorithm performed at various sizes on IonQ devices and simulators. Note that the Forte device does not yet have a corresponding simulator.

In Figure 9, we show the results of these experiments. While IonQ is not explicit about the details of their noise models, we see from Figure 9 that the IonQ simulators predict a roughly linear scaling. This matches the general behavior of the real devices; however, we observe that the Aria simulator consistently under-approximates the observed algorithmic error by a factor of about 2, while the Harmony simulator consistently over-approximates the observed error. The Forte device also exhibits a roughly linear scaling pattern, similar to the Aria device. This indicates two things: first, the IonQ devices scale consistently throughout the “intermediate scale”, and second, IonQ simulators agree with the general behavior of the real devices (up to a scaling factor).

Interestingly, the behavior of the algorithmic error rate for the IBM devices is markedly different from that of IonQ. However, for the complex oracle, neither of the NISQ platforms appears to support real quantum advantage. Assuming that quantum advantage would be present for ∼53 qubits [6], and extrapolating the error rates to such large systems, our results suggest that no studied devices would have an error rate of less than 50% at this scale.

## 5. Concluding Remarks

In conclusion, we performed a benchmarking analysis of the algorithmic error of six NISQ devices using two implementations of Simon’s algorithm. For the most complex oracle, we found that all noisy simulators predict the algorithmic error to scale linearly with the problem size. This prediction was corroborated by the results obtained on IonQ’s trapped-ion NISQ devices. For IBM’s superconducting QPUs, we found that the choice of the oracle function makes an enormous difference in the performance of the algorithm. One crucial source of error was traced back to the sensitivity of CNOT gate operations to the spatial separation of qubits on the hardware.

Our findings have unveiled and re-emphasized several facts about NISQ devices. QPUs based on trapped ions might have some computational advantages due to their all-to-all connectivity. For these devices, less attention needs to be paid to how an algorithm is transpiled onto the hardware. Their obvious shortcoming is still the limited size.

The obvious advantage of superconducting devices is their size and scalability. However, we clearly demonstrated that in the design of algorithms, or rather their transpilation, special attention needs to be paid to the topology of the QPU. For instance, two-qubit operations on spatially separated qubits need to be avoided.

### Future Work

Our work is only the first step of a larger research agenda. As discussed above, Simon’s algorithm is just one representative of the class of hidden subgroup problems. Other examples include the Deutsch–Jozsa algorithm, the Bernstein–Vazirani algorithm, and Shor’s algorithm [2]. The obvious question arises whether our findings are specific to Simon’s algorithm, or whether one would expect similar performance from the other algorithms. All of these algorithms assume access to fault-tolerant qubits, and so studying their behavior on NISQ devices would help to build a more nuanced picture of the current landscape of algorithmic error.

Importantly, such a complete picture would allow for a better understanding of when to expect quantum advantage. One should investigate the amount of error that Simon’s algorithm can tolerate without loss of quantum advantage. The computational advantage of Simon’s algorithm is predicated on the ability to construct a system of n−1 linearly independent values orthogonal to the “secret string” *s*. Such a system can be constructed with fault-tolerant qubits in linear time and then solved classically in polynomial time. With noisy qubits, however, the resulting system of equations will have some equations that are *not* orthogonal to *s*. Hence, the problem becomes solving a noisy system of Boolean linear equations. Some effort has been devoted to researching algorithms for maximally satisfying such systems [57], and a computational complexity result suggesting an exponential scaling for this problem has been derived [58]. Further work is required to establish an exponential scaling for these noisy Boolean linear systems and to determine at what value of algorithmic error the noise overwhelms the advantage of the quantum computation.

Finally, while we can observe the emergence of error in Simon’s algorithm, the physical causes for this error remain unidentifiable with only cloud access. Two approaches could be taken to investigate the origin of noise. First, with sufficiently low-level hardware access, one could probe the algorithm primitives that may cause errors, such as state preparation, measurement, and native hardware gates. Additionally, the relations of these primitives within the algorithm should be explored to characterize the noise phenomena. Alternatively, a sufficiently detailed classical model of the quantum hardware could be constructed. By tuning various noise channels, including depolarizing error, crosstalk error, and state preparation error, one may be able to exactly reconstruct the behavior of the device. The model that succeeds in this reconstruction would shed light on the physical systems from which noise emerges in the real hardware.

## Figures and Tables

**Figure A1:**
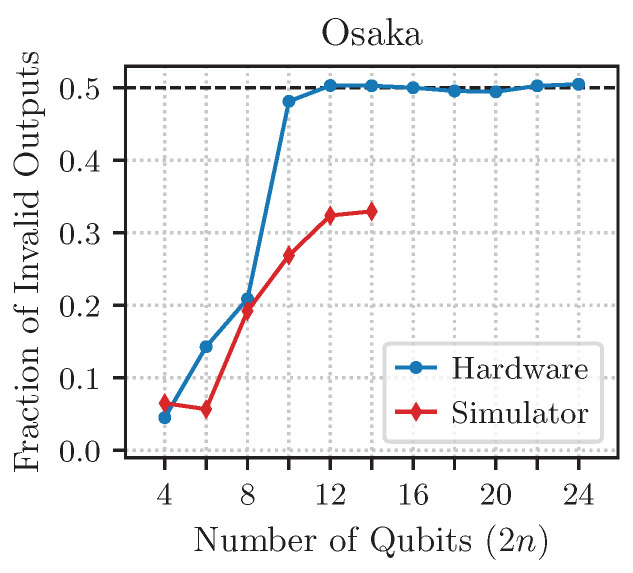
The performance of Simon’s algorithm using the complex oracle, cf. Figure 4a, at various sizes on IBM Osaka and simulator after the major update to Qiskit.

## Data Availability

All code and data for this project can be accessed on GitHub at https://github.com/reecejrobertson/simons-algorithm (accessed on 17 September 2024).

## Abbreviations

The following abbreviations are used in this manuscript:

NISQ	Noisy intermediate scale quantum
QPU	Quantum processing unit
SPAM	State preparation and measurement
XOR	Exclusive-or

## Appendix A. Physical Parameters of the NISQ Devices

In Table A1, we list the physical parameters of the studied NISQ devices. This provides a detailed examination of error sources (including decoherence time, gate errors, and measurement errors) and gives insights into the strengths and weaknesses of each platform.

**Table A1:** Performance metrics for IBM [59] and IonQ [60] devices. Note that for one- and two-qubit (1-Q and 2-Q) gate errors and speeds, as well as state preparation and measurement (SPAM) errors, IBM reports median values while IonQ reports average values. Errors are reported as percentages. The native gate set supported by the IBM devices includes ECR, ID, RZ, SX, and X gates, while the native gate set for the IonQ devices consists of MS, GPI, and GPI2 gates (the Forte device also implements ZZ gates natively [61]).

Parameter	Brisbane	Osaka	Kyoto	Forte	Aria 1	Harmony
Manufacturer	IBM	IBM	IBM	IonQ	IonQ	IonQ
T1 Time	213.12 µs	297.17 µs	215.43 µs	100 s	100 s	10,000 s
T2 Time	145.97 µs	127.23 µs	109.44 µs	1 s	1 s	0.2 s
2-Q Gate Speed	660 ns	660 ns	660 ns	970 µs	600 µs	200 µs
1-Q Gate Error	0.03%	0.03%	0.03%	0.09%	0.06%	0.67%
2-Q Gate Error	0.74%	0.93%	0.92%	0.74%	8.57%	3.07%
Avg SPAM Error	1.32%	2.18%	1.48%	0.5%	0.52%	0.42%
Total Qubits	127	127	127	11	25	36
Topology	Eagle r3	Eagle r3	Eagle r3	all-to-all	all-to-all	all-to-all

While we would like to provide a single number metric to give an idea of the relative efficacy of each device, unfortunately, we cannot. Quantum volume has often been used as a de facto standard for benchmarking devices [37]. However, quantum volume has not been determined for several recent IonQ computers.

Until such time as another single-number metric finds widespread acceptance among the community, we must make do with metrics that can be computed across platforms by individual researchers, such as the algorithmic error studied in this paper.

## Appendix B. Comparison of IBM Device Before and After Update

A rerun of the experiment discussed above was conducted on IBM Osaka, following the major Qiskit and IBM Quantum update of early May 2024. In Figure A1, we show our findings. We see that the performance of both the simulator and real device is comparable to what is shown in Figure 5, with an improvement in the 4–8 qubit range here. Additionally, it is worth noting that after the update, all jobs submitted to Osaka were completed after 5–6 s of runtime, as opposed to the ∼20 s runtime originally observed.
